# Supplementary material for: Dysregulation of the mRNA Expression of Human Renal Drug Transporters by Proinflammatory Cytokines in Primary Human Proximal Tubular Epithelial Cells
Source: Pharmaceutics. 2024 Feb 16;16(2):285. doi: 10.3390/pharmaceutics16020285 (PMC10893102; doi:10.3390/pharmaceutics16020285)
Supplement: Supplementary file 1 [file pharmaceutics-16-00285-s001.zip › pharmaceutics-2779981-supplementary.pdf]

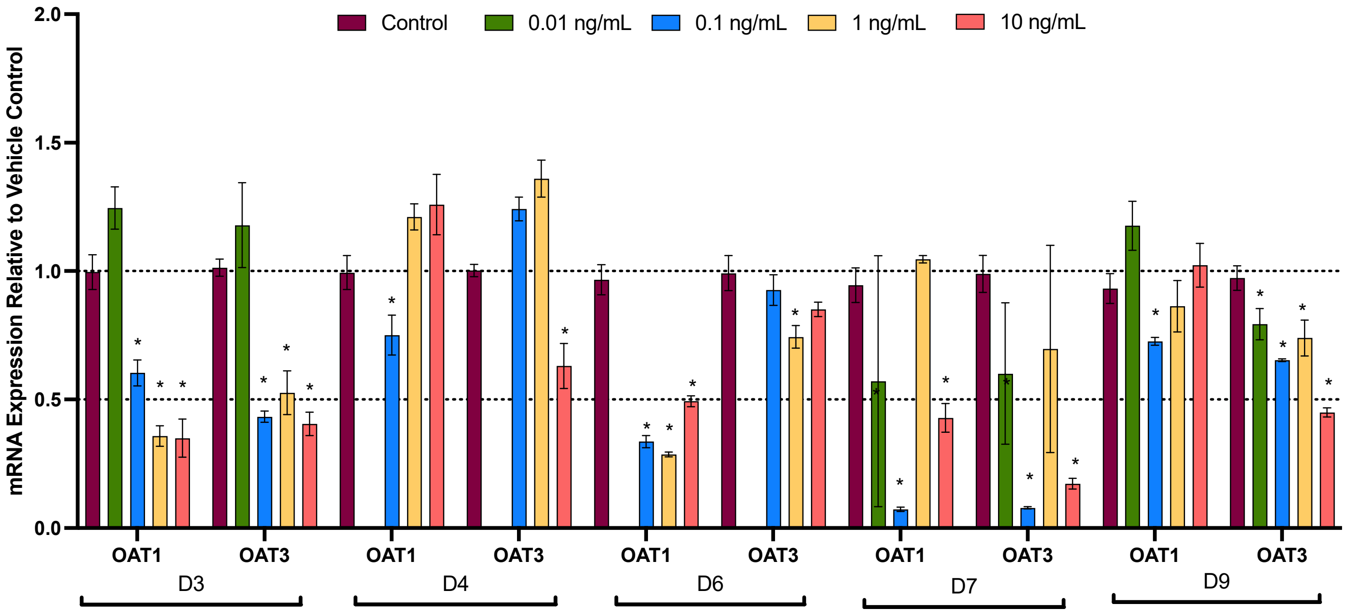

Figure S1. Significant inter-individual (D = donor) variability was observed in the concentration-dependent effect of cytokine cocktails on the mRNA expression of OAT1 and 3 in PTECs. Each cocktail contained all four cytokines at 0.01 (green bar), 0.1 (blue bar), 1 (yellow bar), or 10 ng/mL (orange bar). Data are expressed relative to the mRNA expression in the PTECs treated with the vehicle control (purple bar). Statistical significance (*$p \leq 0.05$) was determined using ANOVA followed by post hoc Fisher's LSD test for pairwise comparisons between the treatment groups and the vehicle control. The quantification of the expression of the gene of interest for each donor was calculated using the comparative C_t value. GAPDH was used as the housekeeping gene. For D4 and D6, 0.01 ng/mL cocktail was not tested due to limited cell yield from kidney tissues.

Table S1. Relative mRNA expression of renal drug transporters in PTECs prior to and after 48 hours of vehicle or cytokine cocktail treatment for donor 3 (D3). The relative expression of transporter genes for each donor was normalized to the expression of the GAPDH, the housekeeping gene. The C_t values of GAPDH in this donor ranges from 20 to 20.5 for all the samples

**Donor**	**Donor 3**					
**Treatment Group / Transporter**	**Prior to treatment**	**48 hours of vehicle treatment**	**48 hours of 0.01 ng/mL cocktail treatment**	**48 hours of 0.1 ng/mL cocktail treatment**	**48 hours of 1 ng/mL cocktail treatment**	**48 hours of 10 ng/mL cocktail treatment**
**Basolateral Transporters**						
OAT1	0.034 ± 0.004	0.0037 ± 0.0002	0.0046 ± 0.0002	0.0022 ± 0.0002	0.0013 ± 0.0001	0.0013 ± 0.0002
OAT3	0.023 ± 0.003	0.00253 ± 0.00007	0.0029 ± 0.0003	0.00108 ± 0.00004	0.0013 ± 0.0002	0.00101 ± 0.00009
OATP4C1	0.347 ± 0.003	0.046 ± 0.001	0.047 ± 0.002	0.0202 ± 0.0003	0.0124 ± 0.0009	0.0082 ± 0.0007
OCT2	0.071 ± 0.002	0.053 ± 0.002	0.0111 ± 0.0007	0.030 ± 0.001	0.0214 ± 0.0004	0.0248 ± 0.0004
**Apical Transporters**						
BCRP	0.0016 ± 0.0005	0.00127 ± 0.00002	0.00044 ± 0.00002	0.00093 ± 0.00009	0.00173 ± 0.00009	0.0016 ± 0.0001
MATE1	0.048 ± 0.003	0.025 ± 0.002	0.0112 ± 0.0004	0.0232 ± 0.0004	0.0220 ± 0.0006	0.0267 ± 0.0008
MATE2-K	0.082 ± 0.001	0.013 ± 0.001	0.00279 ± 0.00005	0.0081 ± 0.0001	0.0048 ± 0.0001	0.0055 ± 0.0001
MRP2	0.011 ± 0.002	0.0085 ± 0.0002	0.00170 ± 0.00003	0.00405 ± 0.00007	0.0026 ± 0.0002	0.003 ± 0.001
MRP3	0.036 ± 0.003	0.033 ± 0.002	0.0174 ± 0.0005	0.042 ± 0.001	0.043 ± 0.002	0.058 ± 0.004
MRP4	0.0210 ± 0.0006	0.0229 ± 0.0004	0.0217 ± 0.0003	0.0154 ± 0.0004	0.0159 ± 0.0007	0.020 ± 0.002
OAT4	0.082 ± 0.004	0.037 ± 0.002	0.00111 ± 0.00001	0.014 ± 0.001	0.0089 ± 0.0002	0.0077 ± 0.0003
OCTN1	0.0025 ± 0.0003	0.00156 ± 0.00004	0.00138 ± 0.00001	0.00250 ± 0.00007	0.0034 ± 0.0002	0.0057 ± 0.0003
OCTN2	0.035 ± 0.002	0.021 ± 0.001	0.0171 ± 0.0004	0.015 ± 0.001	0.0122 ± 0.0002	0.014 ± 0.001
P-gp	0.032 ± 0.004	0.033 ± 0.002	0.027 ± 0.002	0.0223 ± 0.0003	0.0149 ± 0.0003	0.0129 ± 0.0002

Table S2. Relative mRNA expression of renal drug transporters in PTECs prior to and after 48 hours of vehicle or cytokine cocktail treatment for donor 4 (D4). The relative expression of transporter genes for each donor was normalized to the expression of the GAPDH, the housekeeping gene. The C_t values of GAPDH in this donor ranges from 19.5 to 20 for all the samples. N/D, not determined.

Donor	Donor 4					
Treatment Group / Transporter	Prior to treatment	48 hours of vehicle treatment	48 hours of 0.01 ng/mL cocktail treatment	48 hours of 0.1 ng/mL cocktail treatment	48 hours of 1 ng/mL cocktail treatment	48 hours of 10 ng/mL cocktail treatment
**Basolateral Transporters**						
OAT1	0.0032 ± 0.0002	0.00034 ± 0.00004	N/D	0.00026 ± 0.00002	0.00041 ± 0.00001	0.00043 ± 0.00003
OAT3	0.00024 ± 0.00004	0.000021 ± 0.000001	N/D	0.000026 ± 0.000001	0.000028 ± 0.000001	0.000013 ± 0.000001
OATP4C1	0.248 ± 0.008	0.0323 ± 0.0007	N/D	0.028 ± 0.001	0.0200 ± 0.0003	0.0111 ± 0.0003
OCT2	0.0338 ± 0.0005	0.0178 ± 0.0002	N/D	0.0076 ± 0.0002	0.0047 ± 0.0009	0.0040 ± 0.0002
**Apical Transporters**						
BCRP	0.00226 ± 0.00008	0.00156 ± 0.00009	N/D	0.00097 ± 0.00003	0.00092 ± 0.00006	0.00075 ± 0.00005
MATE1	0.0338 ± 0.0001	0.0121 ± 0.0001	N/D	0.0104 ± 0.0005	0.0094 ± 0.0006	0.0106 ± 0.0003
MATE2-K	0.0075 ± 0.0004	0.0012 ± 0.0001	N/D	0.00032 ± 0.00004	0.00016 ± 0.00001	0.0002 ± 0.0001
MRP2	0.0229 ± 0.0007	0.0186 ± 0.0006	N/D	0.0185 ± 0.0007	0.01820 ± 0.00006	0.0201 ± 0.0005
MRP3	0.019 ± 0.002	0.0199 ± 0.0005	N/D	0.0264 ± 0.0008	0.0310 ± 0.0002	0.0294 ± 0.0004
MRP4	0.0013 ± 0.0006	0.00117 ± 0.00006	N/D	0.00080 ± 0.00004	0.00063 ± 0.00002	0.00053 ± 0.00004
OAT4	0.0024 ± 0.0001	0.001515 ± 0.000008	N/D	0.00049 ± 0.00006	0.00039 ± 0.00004	0.00032 ± 0.00002
OCTN1	0.00207 ± 0.00003	0.00121 ± 0.00003	N/D	0.00189 ± 0.00003	0.00273 ± 0.00003	0.0031 ± 0.0001
OCTN2	0.034 ± 0.001	0.0179 ± 0.0004	N/D	0.0183 ± 0.0003	0.0166 ± 0.0002	0.0146 ± 0.0002
P-gp	0.024 ± 0.008	0.0198 ± 0.0003	N/D	0.0180 ± 0.0006	0.012 ± 0.002	0.0083 ± 0.0001

Table S3. Relative mRNA expression of renal drug transporters in PTECs prior to and after 48 hours of vehicle or cytokine cocktail treatment for donor 6 (D6). The relative expression of transporter genes for each donor was normalized to the expression of the GAPDH, the housekeeping gene. The C_t values of GAPDH in this donor ranges from 21 to 22 for all the samples. N/D, not determined.

**Donor**	**Donor 6**					
**Treatment Group / Transporter**	**Prior to treatment**	**48 hours of vehicle treatment**	**48 hours of 0.01 ng/mL cocktail treatment**	**48 hours of 0.1 ng/mL cocktail treatment**	**48 hours of 1 ng/mL cocktail treatment**	**48 hours of 10 ng/mL cocktail treatment**
**Basolateral Transporters**						
OAT1	0.00324 ± 0.00009	0.00049 ± 0.00007	N/D	0.00016 ± 0.00001	0.000140 ± 0.000004	0.000242 ± 0.000008
OAT3	0.0023 ± 0.0004	0.00044 ± 0.00003	N/D	0.00041 ± 0.00002	0.00033 ± 0.00002	0.00037 ± 0.00001
OATP4C1	0.186 ± 0.003	0.014 ± 0.001	N/D	0.011 ± 0.002	0.0067 ± 0.0006	0.006 ± 0.003
OCT2	0.056 ± 0.002	0.036 ± 0.002	N/D	0.020 ± 0.001	0.0132 ± 0.0002	0.0097 ± 0.0008
**Apical Transporters**						
BCRP	0.00071 ± 0.00003	0.00058 ± 0.00005	N/D	0.00067 ± 0.00005	0.00067 ± 0.00004	0.00046 ± 0.00002
MATE1	0.0071 ± 0.0006	0.0048 ± 0.0003	N/D	0.0039 ± 0.0001	0.00367 ± 0.00009	0.0043 ± 0.0002
MATE2-K	0.0081 ± 0.0002	0.0015 ± 0.0001	N/D	0.00070 ± 0.00008	0.00034 ± 0.00002	0.00031 ± 0.00002
MRP2	0.0018 ± 0.0005	0.0016 ± 0.0001	N/D	0.00405 ± 0.00007	0.0026 ± 0.0002	0.003 ± 0.001
MRP3	0.017 ± 0.002	0.0208 ± 0.0010	N/D	0.0262 ± 0.0007	0.0255 ± 0.0005	0.032 ± 0.003
MRP4	0.021 ± 0.002	0.013 ± 0.001	N/D	0.015 ± 0.001	0.0120 ± 0.0004	0.0137 ± 0.0007
OAT4	0.00187 ± 0.00005	0.0015 ± 0.0003	N/D	0.0008 ± 0.0002	0.00045 ± 0.00004	0.000494 ± 0.000008
OCTN1	0.00117 ± 0.00006	0.00084 ± 0.00008	N/D	0.0021 ± 0.0002	0.00177 ± 0.00003	0.0024 ± 0.0002
OCTN2	0.017 ± 0.004	0.0144 ± 0.0009	N/D	0.012 ± 0.001	0.0097 ± 0.0005	0.009 ± 0.004
P-gp	0.0170 ± 0.0008	0.032 ± 0.002	N/D	0.039 ± 0.002	0.037 ± 0.002	0.0252 ± 0.0004

Table S4. Relative mRNA expression of renal drug transporters in PTECs prior to and after 48 hours of vehicle or cytokine cocktail treatment for donor 7 (D7). The relative expression of transporter genes for each donor was normalized to the expression of the GAPDH, the housekeeping gene. The C_t values of GAPDH in this donor ranges from 17.5 to 18 for all the samples.

Donor	Donor 7					
Treatment Group / Transporter	Prior to treatment	48 hours of vehicle treatment	48 hours of 0.01 ng/mL cocktail treatment	48 hours of 0.1 ng/mL cocktail treatment	48 hours of 1 ng/mL cocktail treatment	48 hours of 10 ng/mL cocktail treatment
**Basolateral Transporters**						
OAT1	0.00055 ± 0.00007	0.000051 ± 0.000005	0.000017 ± 0.000004	0.0000037 ± 0.0000004	0.000054 ± 0.000001	0.000020 ± 0.000002
OAT3	0.00086 ± 0.00002	0.000054 ± 0.000009	0.000023 ± 0.000001	0.0000042 ± 0.000002	0.00004 ± 0.00002	0.0000085 ± 0.0000008
OATP4C1	0.13 ± 0.03	0.0150 ± 0.0007	0.0158 ± 0.0002	0.0147 ± 0.0003	0.0127 ± 0.0004	0.008 ± 0.003
OCT2	0.0124 ± 0.0003	0.0071 ± 0.0004	0.00463 ± 0.00005	0.00295 ± 0.00003	0.00225 ± 0.00007	0.00193 ± 0.00009
**Apical Transporters**						
BCRP	0.00056 ± 0.00006	0.00044 ± 0.00002	0.000280 ± 0.000002	0.000240 ± 0.000009	0.00021 ± 0.00002	0.000144 ± 0.000005
MATE1	0.0045 ± 0.0004	0.00134 ± 0.00007	0.001092 ± 0.000005	0.001277 ± 0.000006	0.00196 ± 0.00002	0.00137 ± 0.00002
MATE2-K	0.000232 ± 0.000009	0.000049 ± 0.000001	0.000037 ± 0.000002	0.000005 ± 0.000001	0.000008 ± 0.000002	0.000007 ± 0.000002
MRP2	0.0010 ± 0.0001	0.000218 ± 0.000005	0.000172 ± 0.000006	0.000152 ± 0.000009	0.000103 ± 0.000005	0.000151 ± 0.000009
MRP3	0.0068 ± 0.0005	0.0034 ± 0.0005	0.00492 ± 0.00003	0.0081 ± 0.0002	0.0135 ± 0.0006	0.0169 ± 0.0005
MRP4	0.0151 ± 0.0006	0.0129 ± 0.0005	0.0099 ± 0.0004	0.0075 ± 0.0002	0.0089 ± 0.0002	0.0095 ± 0.0004
OAT4	0.000078 ± 0.000004	0.000047 ± 0.000002	0.000030 ± 0.000002	0.0000251 ± 0.0000009	0.0000164 ± 0.0000007	0.000016 ± 0.000004
OCTN1	0.00077 ± 0.00008	0.000316 ± 0.000003	0.000403 ± 0.000002	0.000601 ± 0.000006	0.00119 ± 0.00006	0.00118 ± 0.00004
OCTN2	0.0124 ± 0.0004	0.0087 ± 0.0004	0.0063 ± 0.0002	0.0072 ± 0.0001	0.0092 ± 0.0004	0.0080 ± 0.0003
P-gp	0.072 ± 0.0002	0.0055 ± 0.0002	0.00510 ± 0.00007	0.0052 ± 0.0002	0.0032 ± 0.0001	0.00281 ± 0.00006

Table S5. Relative mRNA expression of renal drug transporters in PTECs prior to and after 48 hours of vehicle or cytokine cocktail treatment for donor 9 (D9). The relative expression of transporter genes for each donor was normalized to the expression of the GAPDH, the housekeeping gene. The C_t values of GAPDH in this donor ranges from 18 to 18.5 for all the samples.

**Donor**	**Donor 9**					
**Treatment Group / Transporter**	**Prior to treatment**	**48 hours of vehicle treatment**	**48 hours of 0.01 ng/mL cocktail treatment**	**48 hours of 0.1 ng/mL cocktail treatment**	**48 hours of 1 ng/mL cocktail treatment**	**48 hours of 10 ng/mL cocktail treatment**
**Basolateral Transporters**						
OAT1	0.00032 ± 0.00003	0.000050 ± 0.000004	0.000059 ± 0.000004	0.0000362 ± 0.0000006	0.000043 ± 0.000004	0.00005 ± 0.00004
OAT3	0.0031 ± 0.0002	0.00024 ± 0.00002	0.00019 ± 0.00001	0.000157 ± 0.000001	0.00018 ± 0.00001	0.00011 ± 0.00004
OATP4C1	0.228 ± 0.004	0.0226 ± 0.0007	0.014 ± 0.001	0.0126 ± 0.0004	0.0066 ± 0.0001	0.00157 ± 0.00009
OCT2	0.022 ± 0.004	0.0125 ± 0.0006	0.0097 ± 0.0004	0.0076 ± 0.0006	0.0060 ± 0.0002	0.0037 ± 0.0002
**Apical Transporters**						
BCRP	0.00030 ± 0.00004	0.00022 ± 0.00001	0.000329 ± 0.000008	0.00029 ± 0.00002	0.00030 ± 0.00001	0.00017 ± 0.00001
MATE1	0.0053 ± 0.0006	0.0020 ± 0.0001	0.00036 ± 0.00009	0.00200 ± 0.00003	0.00239 ± 0.00001	0.0021 ± 0.0001
MATE2-K	0.0051 ± 0.0004	0.0013 ± 0.00004	0.00152 ± 0.00006	0.00063 ± 0.00001	0.00055 ± 0.00002	0.00027 ± 0.00003
MRP2	0.0014 ± 0.0007	0.00113 ± 0.00008	0.00117 ± 0.00005	0.00085 ± 0.00005	0.00058 ± 0.00002	0.00050 ± 0.00004
MRP3	0.0136 ± 0.0003	0.0130 ± 0.0006	0.0210 ± 0.0009	0.0319 ± 0.0007	0.053 ± 0.006	0.038 ± 0.005
MRP4	0.0093 ± 0.0005	0.0075 ± 0.0004	0.0087 ± 0.0004	0.0081 ± 0.0004	0.0064 ± 0.0004	0.0043 ± 0.0001
OAT4	0.0063 ± 0.0006	0.0037 ± 0.0002	0.0040 ± 0.0001	0.0022 ± 0.0001	0.0020 ± 0.0002	0.0020 ± 0.0001
OCTN1	0.00041 ± 0.00006	0.00027 ± 0.00001	0.00037 ± 0.00001	0.00069 ± 0.00006	0.001133 ± 0.000005	0.00150 ± 0.00006
OCTN2	0.0092 ± 0.0005	0.0066 ± 0.0002	0.0087 ± 0.0004	0.0071 ± 0.0004	0.0060 ± 0.0004	0.0037 ± 0.0004
P-gp	0.00390 ± 0.00004	0.0042 ± 0.0002	0.0068 ± 0.0001	0.0036 ± 0.0001	0.00244 ± 0.00009	0.00141 ± 0.00003
